# A meta-analysis of the combined effects of elevated carbon dioxide and chronic warming on plant %N, protein content and N-uptake rate

**DOI:** 10.1093/aobpla/plab031

**Published:** 2021-05-25

**Authors:** Dileepa M Jayawardena, Scott A Heckathorn, Jennifer K Boldt

**Affiliations:** 1 Department of Environmental Sciences, University of Toledo, Toledo, OH 43606, USA; 2 Agricultural Research Service, United States Department of Agriculture, Toledo, OH 43606, USA

**Keywords:** Climate change, elevated CO_2_, heat stress, meta-analysis, nitrogen metabolism, nitrogen translocation, nitrogen-uptake rate, protein, warming

## Abstract

Elevated CO_2_ (eCO_2_) and high temperatures are known to affect plant nitrogen (N) metabolism. Though the combined effects of eCO_2_ and chronic warming on plant N relations have been studied in some detail, a comprehensive statistical review on this topic is lacking. This meta-analysis examined the effects of eCO_2_ plus warming on shoot and root %N, tissue protein concentration (root, shoot and grain) and N-uptake rate. In the analyses, the eCO_2_ treatment was categorized into two classes (<300 or ≥300 ppm above ambient or control), the temperature treatment was categorized into three classes (<1.5, 1.5–5 and >5 °C above ambient or control), plant species were categorized based on growth form and functional group and CO_2_ treatment technique was also investigated. Elevated CO_2_ alone or in combination with warming reduced shoot %N (more so at ≥300 vs. <300 ppm above ambient CO_2_), while root %N was significantly reduced only by eCO_2_; warming alone often increased shoot %N, but mostly did not affect root %N. Decreased shoot %N with eCO_2_ alone or eCO_2_ plus warming was greater for woody and non-woody dicots than for grasses, and for legumes than non-legumes. Though root N-uptake rate was unaffected by eCO_2_, eCO_2_ plus warming decreased N-uptake rate, while warming alone increased it. Similar to %N, protein concentration decreased with eCO_2_ in shoots and grain (but not roots), increased with warming in grain and decreased with eCO_2_ and warming in grain. In summary, any benefits of warming to plant N status and root N-uptake rate will generally be offset by negative effects of eCO_2_. Hence, concomitant increases in CO_2_ and temperature are likely to negate or decrease the nutritional quality of plant tissue consumed as food by decreasing shoot %N and shoot and/or grain protein concentration, caused, at least in part, by decreased root N-uptake rate.

## Teaser

In the future, concomitant increases in CO_2_ and temperature are likely to affect plant N metabolism by lowering plant %N, root N-uptake rate and tissue protein concentration. In addition, different growth forms will respond differently to concomitant increases in CO_2_ and temperature. Elevated CO_2_ plus warming is likely to enhance root-to-shoot N translocation in grasses but decrease it in non-woody dicots and woody species. Further, in addition to the FACE technique, other enclosed techniques such as GC, TGT and CTC can produce reliable results for studies examining the effects of eCO_2_ plus warming.

## Introduction

Present-day atmospheric carbon dioxide (CO_2_) levels (ca. 400 ppm) are unprecedented over the past 420 000 years (Petit *et al.* 1999). With industrialization, and its expansion due to economic and population growth, atmospheric CO_2_ levels have increased up to 46 % in the last 170 years. According to low and intermediate CO_2_-emission scenarios, atmospheric CO_2_ is likely to be in the range of 450–1000 ppm by the end of this century (IPCC 2014). Carbon dioxide is a greenhouse gas and its emissions account for ca. two-thirds of current global warming. Due to global warming, the increase in Earth’s mean surface temperature is likely to be in the range of 1.5–6 °C by 2100 (IPCC 2014). The concomitant increases in CO_2_ and temperature are expected to have various impacts on plant species, including those used in agriculture and forestry. Though the interactive effects of CO_2_ enrichment and warming on plant growth and function have been studied in some detail (Morison and Lawlor 1999; Wang *et al.* 2012), nitrogen (N) metabolism in response to concomitant increases in CO_2_ and temperature is still poorly understood.

Plant N relations in response to eCO_2_ have been extensively studied. Due to natural variation among species and differences among experimental protocols, most plant responses to eCO_2_ are highly variable; one exception to this pattern is tissue N concentration (Bassirirad 2000; Ainsworth and Long 2005; Taub and Wang 2008; Ainsworth and Long 2021). Elevated CO_2_ stimulates photosynthesis which then stimulates production of non-structural carbohydrates. When the sugar production exceeds the plant sink capacity, it induces a negative feedback on the transcription of Ribulose-1,5-bisphosphate carboxylase-oxygenase (Rubisco), resulting in reduced Rubisco concentrations (and thus leaf N; see also Moore *et al.* 1999), and thus net CO_2_ assimilation (which may still be higher compared to plants grown under ambient CO_2_) (Ainsworth and Long 2005; Taub and Wang 2008). According to the progressive N limitation (PNL) hypothesis, eCO_2_ could enhance the sequestration of N into long-lived plant biomass and soil organic matter which in turn reduce the available soil N for plant growth resulting lower tissue N (Luo *et al*. 2004). In addition, several other mechanisms, such as decreased root N uptake, less efficient root architecture and increased N loss, have been proposed to contribute to lower tissue N concentrations at eCO_2_ (Taub and Wang 2008). As with tissue %N, eCO_2_ is also likely to reduce tissue protein concentration (Taub *et al.* 2008; Feng *et al.* 2015). However, legumes are hypothesized to have an advantage over other C_3_ species when grown at eCO_2_ due to their ability to exchange carbon for N with their N-fixing symbionts (Rogers *et al.* 2009). Though eCO_2_ tends to decrease root N-uptake rate, especially in plants rooted in solid media, past studies collectively have shown that both N-uptake kinetics and root N-uptake rate in response to eCO_2_ can be highly variable (Bassirirad 2000; Taub and Wang 2008).

Plant N relations in response to chronic warming have been studied in some detail. A meta-analysis conducted by Zvereva and Kozlov (2006) found a non-significant negative effect of warming (≥4 or <4 °C above control) on above-ground N concentration. Heat stress is known to cause rates of protein degradation to exceed rates of new protein synthesis (Huang *et al.* 2012). Huang *et al.* (2012) further showed that some cultivars were capable of producing more thermostable proteins and maintaining low levels of proteolytic enzyme activities to protect against warming. Therefore, though warming is likely to reduce protein concentration, variation in plant protein levels in response to warming could be either interspecies- or intraspecies-specific. Optimal growth temperature is species-specific, and hence, warming from suboptimal to optimal temperatures is likely to increase root N-uptake rate (Clarkson and Warner 1979; Tindall *et al.* 1990; Cruz *et al.* 1993; Atkin and Cummins 1994), while warming (acute or chronic) from optimal to supra-optimal temperatures is likely to decrease this rate (Tindall *et al.* 1990; Delucia *et al.* 1992; Bassirirad *et al.* 1993; Mainali *et al.* 2014; Giri *et al.* 2017).

Combined eCO_2_ plus warming is likely to decrease above-ground N concentration in plant tissue, and the magnitude of this decrease has been greater for C_3_ and woody species than C_4_ and herbaceous species (Zvereva and Kozlov 2006; Wang *et al.* 2012); however, root %N is likely to be unaffected by eCO_2_ plus warming (Wang *et al.* 2012). A limited number of studies suggests that the effects of eCO_2_ plus warming on N-uptake rate can be variable, within and among species (Coleman and Bazzaz 1992; Dijkstra *et al.* 2010; Arndal *et al*. 2014; Jayawardena *et al.* 2017). Few studies have looked at the effects of eCO_2_ plus warming on tissue protein concentration (total protein per g dry mass). They suggest that grain protein concentration can vary in response to eCO_2_ plus warming (Abebe *et al.* 2016; Jing *et al.* 2016; Palacios *et al.* 2019; Qiao *et al.* 2019). Previously, we examined the effects of eCO_2_ plus warming on total root protein concentration of tomato (*Solanum lycopersicum*) provided either nitrate (NO_3_^−^) or ammonium (NH_4_^+^) as the sole N source and noted a significant decrease in root protein concentration in both sets of plants (Jayawardena *et al.* 2017). To the best of our knowledge, total shoot protein concentration in response to eCO_2_ plus warming has not been studied before. Collectively, these studies indicate that the effects of eCO_2_ plus warming on root N uptake and assimilation are not fully understood.

Free-air CO_2_ enrichment (FACE) experiments are thought to provide the most realistic measures of the effects of eCO_2_ on crop yields (Ainsworth *et al.* 2008; Ainsworth and Long 2021). However, the high cost associated with FACE experiments (ca. US$1 million in maintenance per year plus investigation costs) (DaMatta *et al.* 2010) makes it inaccessible to many researchers who are interested in investigating environmental variables such as CO_2_ and temperature on plant responses. Though many available enclosure techniques produce a ‘chamber effect’ (Ainsworth *et al.* 2008), they have often produced results similar to those of FACE experiments (e.g., see Taub *et al.* 2008). This comparison has mostly been assessed only for eCO_2_ and not eCO_2_ plus other factors, so it would be useful to compare plant responses (e.g. plant %N) to eCO_2_ plus warming using different eCO_2_ treatment delivery techniques.

Our current understanding of the effects of eCO_2_ plus warming on plant N metabolism has important knowledge gaps. Therefore, the main objective of this study was to narrow these knowledge gaps using a comprehensive meta-analysis of the effects of eCO_2_ plus warming on variables related to plant N metabolism, such as shoot and root %N, tissue protein concentration and root N-uptake rate. This includes a subgroup analysis of the effects of eCO_2_ plus warming on different growth forms, functional groups and eCO_2_ treatment techniques. Results of this study will help crop scientists, plant breeders and molecular biologists better understand how plant N metabolism will likely respond to future predicted climate conditions and provide targets for developing new genotypes with improved N-relation traits suited for future climate conditions.

## Methods

### Data collection

This meta-analysis mostly followed methods described by Wang *et al.* (2012). A literature search was conducted between November 2019 and March 2020 using the search engines PubMed and Google Scholar to construct the database for this meta-analysis (Table 1). The obtained peer-reviewed research or review papers were cross-referenced to help ensure the inclusion of all relevant articles. Research papers published in English which met the following criteria were included in the meta-analysis: (i) CO_2_ × temperature treatment interaction (full factorial or at least the interaction and the control treatment), (ii) warming treatment that was chronic (i.e. growing plants for a longer period of time ranging from weeks to years at higher than ambient/near-optimal temperatures; abrupt or short-term heating was not considered here, i.e. heat-shocking plants with supra-optimal temperatures for hours to a few days), (iii) whole-plant warming throughout the day (soil or night-time-only warming was excluded) and (iv) reporting of standard error or standard deviation and number of replicates. For all studies considered, ambient or control CO_2_ concentration (aCO_2_) ranged between 350 and 400 ppm, while eCO_2_ ranged between 490 and 800 ppm. If aCO_2_ was not reported for a study, mean annual CO_2_ concentration for the year in which the study was conducted was estimated using CO_2_.earth (https://www.co2.earth/monthly-co2). The lowest elevated temperature was ambient temperature (*T*_amb_) + 0.6 °C, while the highest elevated temperature was *T*_amb_ + 19 °C. However, there were only four experimental observations with elevated temperatures >10 °C above ambient. Response variables extracted for analysis included shoot and root %N (per unit dry mass), root N-uptake rate (rate per unit dry mass per unit time) and tissue protein concentration (total protein per unit dry mass). Shoot %N data had four experimental observations with partial irrigation, eight experimental observations with insect feeding and one experimental observation with elevated Ultraviolet-B light treatment. Root %N data had one experimental observation with partial irrigation. When taking these observations into account, the non-CO_2_ or non-temperature-stress treatment alone was used as the control. These observations were not excluded from the database in order to increase sample size (and response patterns did not change with the exclusion of these observations). Root N-uptake rate included total N, NO_3_^−^ or NH_4_^+^ uptake rates of intact or excised roots. Graphically presented data were extracted using the data extraction software WebPlotDigitizer version 4.2 (Rohatgi 2017). Standard errors of the mean (SEM) were converted to standard deviations (SDs) using the equation; SD=SEM×√n, where *n* is the number of replicates **[see****Supporting Information—Appendix S1****]**.

**Table 1.** Plant species, response variables extracted, subgroup of the response variable extracted and references used in the meta-analysis.

Species	Response variable extracted				Subgroup of the response variable extracted			References
	Shoot %N	Root %N	Root N-uptake rate	Protein concentration	Growth form	Functional group	Treatment technique	
*Abutilon theophrasti*	*	*			NWD	NL	GC	Coleman and Bazzaz (1992)
*Acer rubrum*	*				W	NL	OTC	Norby *et al.* (2000)
*Acer rubrum*		*			W	NL	OTC	Wan *et al.* (2004)
*Acer rubrum*	*				W	NL	OTC	Williams *et al.* (2003)
*Acer rubrum*	*				W	NL	OTC	Williams *et al.* (2000)
*Acer saccharum*	*				W	NL	OTC	Norby *et al.* (2000)
*Acer saccharum*		*			W	NL	OTC	Wan *et al.* (2004)
*Acer saccharum*	*				W	NL	OTC	Williams *et al.* (2000)
*Alliaria petiolata*	*				NWD	NL	GC	Anderson and Cipollini (2013)
*Amaranthus retroflexus*	*	*			NWD	NL	GC	Coleman and Bazzaz (1992)
*Betula pendula*			*		W	NL	GC	Kellomaki and Wang (2001)
*Betula pendula*	*				W	NL	CTC	Kuokanen *et al*. (2001)
*Betula pendula*	*				W	NL	CTC	Kuokanen *et al*. (2003)
*Betula pendula*	*				W	NL	CTC	Lavola *et al.* (2013)
*Brassica juncea*	*				NWD	NL	GC	Seth and Misra (2014)
*Calluna vulgaris*	*	*			W	NL	FACE	Andresen *et al.* (2009)
*Calluna vulgaris*	*	*			W	NL	FACE	Andresen *et al.* (2010)
*Calluna vulgaris*			*		W	NL	FACE	Arndal *et al*. (2014)
*Coffea arabica*	*				G	NL	GC	Ramalho *et al.* (2018)
*Deschampsia flexuosa*	*	*			W	NL	FACE	Andresen *et al.* (2009)
*Deschampsia flexuosa*	*	*			G	NL	FACE	Andresen *et al.* (2010)
*Deschampsia flexuosa*			*		G	NL	FACE	Arndal *et al*. (2014)
*Echinium plantagineum*	*				NWD	NL	GC	Johns and Hughes (2002)
*Eucalyptus globulus*	*				W	NL	CTC	Crous *et al.* (2013)
*Eucalyptus globulus*	*				W	NL	OTC	Sharwood *et al.* (2017)
*Eucalyptus robusta*	*				W	NL	GH	Gherlenda *et al.* (2015)
*Eucalyptus saligna*	*				W	NL	GH	Ayub *et al.* (2011)
*Eucalyptus saligna*	*				W	NL	GH	Ghannoum *et al.* (2010a)
*Eucalyptus saligna*	*	*			W	NL	GH	Ghannoum *et al*. (2010b)
*Eucalyptus sideroxylon*	*				W	NL	GH	Ghannoum *et al.* (2010a)
*Eucalyptus sideroxylon*	*	*			W	NL	GH	Ghannoum *et al*. (2010b)
*Eucalyptus tereticornis*	*				W	NL	GH	Gherlenda *et al.* (2015)
*Eucalyptus tereticornis*	*				W	NL	GH	Gherlenda *et al.* (2016)
*Eucalyptus tereticornis*	*				W	NL	GH	Murray *et al.* (2013)
*Geum vernum*	*				NWD	NL	GC	Anderson and Cipollini (2013)
*Glycine max*				*	NWD	L	OTC	Palacios *et al.* (2019)
*Glycine max*				*	NWD	L	OTC	Qiao *et al.* (2019)
*Glycine max*	*				NWD	L	FACE	Rosenthal *et al.* (2014)
*Gossypium hirsutum*	*				W	NL	GC	Zhang *et al.* (2017)
Grasses	*				G	NL	GC	Johnson and Hartley (2018)
*Lantana camara*	*				W	NL	GC	Johns *et al.* (2003)
*Lolium perenne*	*	*			G	NL	CTC	Soussana *et al.* (1996)
*Lolium perenne*	*				G	NL	CTC	Zavalloni *et al.* (2012)
*Lotus corniculatus*	*				NWD	L	CTC	Zavalloni *et al.* (2012)
*Medicago lupulina*	*				NWD	L	CTC	Zavalloni *et al.* (2012)
*Medicago sativa*	*				NWD	L	TGT	Aranjuelo *et al.* (2005)
*Medicago sativa*		*			NWD	L	TGT	Aranjuelo *et al.* (2008)
*Medicago sativa*	*				NWD	L	GH	Ariz *et al*. (2015)
*Oryza sativa*				*	G	NL	FACE	Jing *et al.* (2016)
*Oryza sativa*	*				G	NL	TGT	Kim *et al.* (2011)
*Oryza sativa*	*	*			G	NL	FACE	Li *et al.* (2017)
*Oryza sativa*	*				G	NL	OTC	Liu *et al.* (2019)
*Panicum maximum*	*				G	NL	FACE	de Assis Prado *et al*. (2016)
*Phalaris aquatica*	*				G	NL	TGT	Lilley *et al.* (2001)
*Phalaris aquatica*	*				G	NL	TGT	Volder *et al*. (2015)
*Phaseolus vulgaris*	*				NWD	L	CTC	Prasad *et al*. (2004)
*Pinus ponderosa*		*			W	NL	GH	King *et al.* (1997)
*Pinus sylvestris*	*				W	NL	CTC	Luomala *et al.* (2003)
*Pinus taeda*		*			W	NL	GH	King *et al.* (1997)
*Plantago lanceolata*	*				G	NL	CTC	Zavalloni *et al.* (2012)
*Poa pratensis*	*				G	NL	CTC	Zavalloni *et al.* (2012)
*Pseudotsuga menziesii*		*			W	NL	CTC	Chen *et al.* (2008)
*Pseudotsuga menziesii*	*				W	NL	CTC	Hobbie *et al.* (2001)
*Quercus robur*	*				W	NL	GH	Dury *et al.* (1998)
*Rumex acetosa*	*				NWD	NL	CTC	Zavalloni *et al.* (2012)
*Salix myrsinifolia*	*				W	NL	CTC	Veteli *et al.* (2002)
*Semiarid grasses*	*	*			G	NL	FACE	Dijkstra *et al.* (2010)
*Solanum lycopersicum*	*	*	*	*	NWD	NL	GC	Jayawardena *et al.* (2017)
*Solanum lycopersicum*	*	*	*	*	NWD	NL	GC	Jayawardena *et al.* (2021)
Species mix		*			N/A	NL	CTC	Kandeler *et al.* (1998)
Species mix	*				N/A	NL	FACE	Mueller *et al.* (2016)
Species mix		*			G	NL	CTC	Zavalloni *et al.* (2012)
*Trifolium subterraneum*	*				NWD	L	TGT	Lilley *et al.* (2001)
*Triticum aestivum*	*	*	*	*	G	NL	GC	Jayawardena *et al.* (2020)
*Triticum durum*	*				NWD	NL	TGT	Jauregui *et al.* (2015)
*Vitis vinifera*	*	*			W	NL	GH	Salazar-Parra *et al.* (2015)
*Zea mays*				*	G	NL	OTC	Abebe *et al.* (2016)
*Zea mays*	*				G	NL	CTC	Kim *et al.* (2007)
*Zea mays*				*	G	NL	OTC	Qiao *et al.* (2019)

References included in the meta-analysis are available in Supporting Information—Appendix S2.

* denotes the response variable extracted from each reference. Different growth forms are denoted as woody, W; grassy, G; and non-woody dicot, NWD. Different functional groups are denoted as legume, L; and non-legume, NL. Different treatment techniques are denoted as open-top chambers, OTC; closed-top chambers, CTC; greenhouses, GH; free-air CO_2_ enrichment, FACE; temperature-gradient tunnels, TGT; and growth chambers, GC.

### Categorization of data

The increase in global mean surface temperature is likely to exceed 1.5 °C by 2100 under all emission scenarios. It is also likely to be in the range of 1.5–4.5 °C, and very unlikely to be greater than 6 °C, by 2100 (IPCC 2014). Based on these predictions, the warming treatments were categorized into three temperature classes as ambient or control plus: <1.5 °C (*T*_L_), 1.5–5 °C (*T*_M_) and >5 °C (*T*_H_). According to the low and intermediate CO_2_ emission scenarios, atmospheric CO_2_ is likely to increase between 450 and 1000 ppm by 2100 (IPCC 2014). Therefore, a breakpoint for categorization of eCO_2_ treatments into two levels was arbitrarily selected as <300 ppm or ≥300 ppm above ambient or control (350–400 ppm CO_2_). Plant species were categorized based on growth form (woody, grassy or non-woody dicots), functional group (legumes or non-legumes) and treatment technique (open-top chambers, OTC; closed-top chambers, CTC; greenhouses, GH; FACE; temperature-gradient tunnels, TGT; or growth chambers, GC). Only shoot and root %N data were categorized into temperature or CO_2_ subclasses (growth form, functional group and treatment technique) due to the high availability of experimental observations for these two response variables. Protein concentration was categorized based on tissue type (grain, shoot or root).

### Meta-analytic method

In this meta-analysis, the natural-log response ratio [ln(r)] between the means of experimental and control groups was used as the metric of the effect size (Hedges *et al.* 1999). The effect size was graphically presented as the mean % change [(r−1)×100] (Ainsworth *et al.* 2002) with its 95 % confidence interval (CI). The meta-analysis was performed using OpenMEE an open-source software for meta-analysis in ecology and evolutionary biology (Wallace *et al.* 2017). A continuous random-effects model with Hedges–Olkin method that relies on inverse-variance weighting to account for variation in precision (sampling error) within and between studies was used (Hedges and Olkin 1985; Wallace *et al.* 2017). The independent variables (eCO_2_, warming and eCO_2_ plus warming) were considered to have a significant effect on the dependent variables if CIs did not overlap the reference line at 0 % change. The outcome was considered significant if *P* < 0.05. Normality assumption was checked using normal quantile–quantile plots. Publication bias was checked using Rosenthal’s fail-safe number and funnel plots. Rosenthal’s fail-safe number was calculated using OpenMEE software. This number indicates the number of non-significant and unpublished studies required for the meta-analysis to change the statistical significance of the meta-analytic result to a non-significant result (Rosenthal 1979). If this number was greater than 5n+10, where *n* is the number of experimental observations, publication bias could be safely ignored (Rosenberg 2005). In addition, if data were symmetrically distributed in the funnel plot, publication bias was safely ignored.

## Results

**Figure 1.**
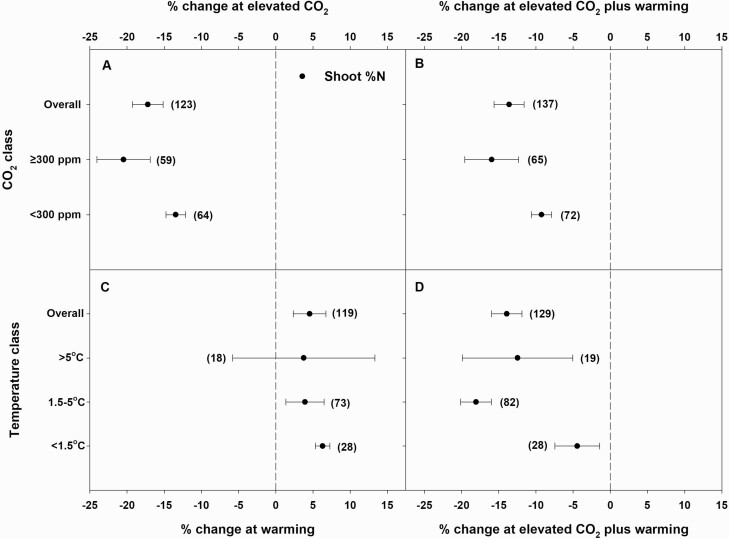
Percent change (compared to ambient or controls) in shoot %N in response to elevated CO_2_ (eCO_2_) (A) or eCO_2_ plus warming (B) at different eCO_2_ classes (ambient + <300 or ≥300 ppm) and warming (C) or eCO_2_ plus warming (D) at different temperature classes (ambient + <1.5, 1.5–5 or >5 °C). Each data point represents the mean ± 95 % CI. Numbers within parentheses represent the number of experimental observations. The dashed vertical line is the reference line at 0 % change. Treatment effects are non-significant at *P* < 0.05 if CIs overlap the zero line, and differences among treatments are non-significant if CIs overlap.

**Figure 2.**
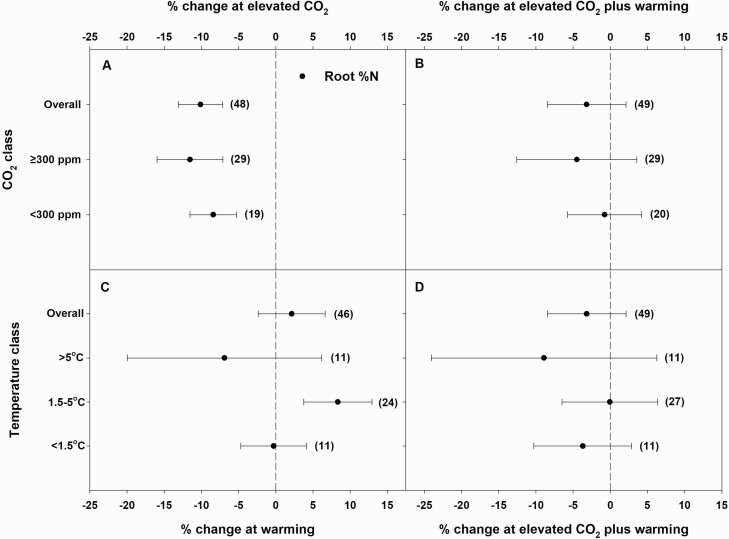
Percent change (compared to ambient or controls) in root %N in response to elevated CO_2_ (eCO_2_) (A) or eCO_2_ plus warming (B) at different eCO_2_ classes (ambient + <300 or ≥300 ppm) and warming (C) or eCO_2_ plus warming (D) at different temperature classes (ambient + <1.5, 1.5–5 or >5 °C). Each data point represents the mean ± 95 % CI. Numbers within parentheses represent the number of experimental observations. The dashed vertical line is the reference line at 0 % change. Treatment effects are non-significant at *P* < 0.05 if CIs overlap the zero line, and differences among treatments are non-significant if CIs overlap.

Elevated CO_2_ alone significantly reduced both shoot and root %N, by 17 and 10 % overall, respectively. The magnitude of the decrease was greater at high eCO_2_ (≥300 ppm above control) than at low eCO_2_ (<300 ppm above control), although it was significant only for shoots (Figs 1A and 2A). Warming alone significantly increased shoot %N by 5 % and non-significantly increased root %N by 2 %. The magnitude of a warming-driven increase in shoot %N decreased as temperature increases from *T*_L_ to *T*_H_, although CIs overlapped for all three temperature classes. Notably, the increase in shoot %N at *T*_H_ was non-significant (Fig. 1C). Meanwhile, root %N neither increased nor decreased at *T*_L_, but significantly increased at *T*_M_ and non-significantly decreased at *T*_H_ (Fig. 2C). There was a publication bias for the effect of temperature on root %N. Irrespective of the CO_2_ or temperature classes, eCO_2_ plus warming significantly reduced shoot %N by 14 %. The magnitude of decrease was greater at high eCO_2_ or *T*_M_ (1.5–5 °C above control) than at low eCO_2_ or *T*_L_ (<1.5 °C above control) (Fig. 1B and D). Elevated CO_2_ plus warming non-significantly decreased root %N by 3 % and trended towards a greater decrease at high eCO_2_ or *T*_H_ (>5 °C above control) than at low eCO_2_ or *T*_M_ (Fig. 2B and D). There was a publication bias for the effects eCO_2_ plus warming on root %N.

**Figure 3.**
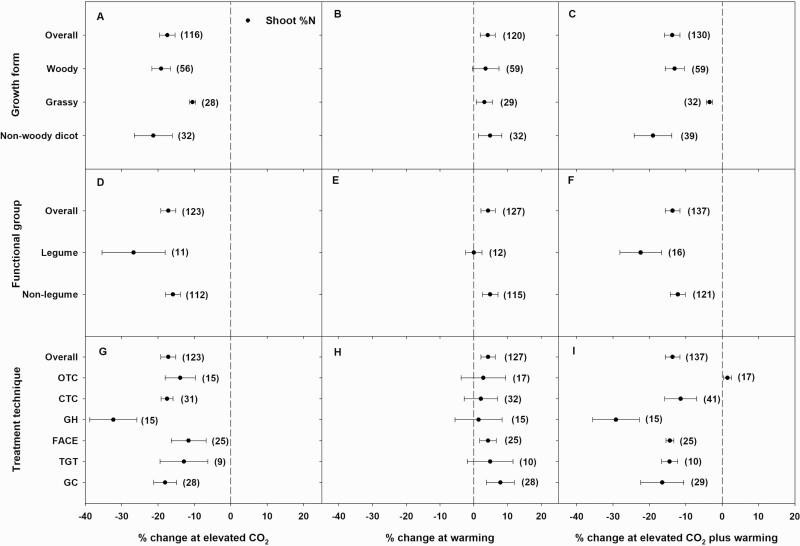
Percent change (compared to ambient or controls) in shoot %N in different growth forms (A–C), functional groups (D–F) and elevated CO_2_ (eCO_2_) treatment techniques (G–I, OTC = open-top chambers; CTC = closed-top chambers; GH = greenhouses; FACE = free-air CO_2_ enrichment; TGT = temperature-gradient tunnels; GC = growth chambers) in response to eCO_2_ (A, D, G), warming (B, E, H) and eCO_2_ plus warming (C, F, I). Each data point represents the mean ± 95 % CI. Numbers within parentheses represent the number of experimental observations. The dashed vertical line is the reference line at 0 % change. Treatment effects are non-significant at *P* < 0.05 if CIs overlap the zero line, and differences among treatments are non-significant if CIs overlap.

**Figure 4.**
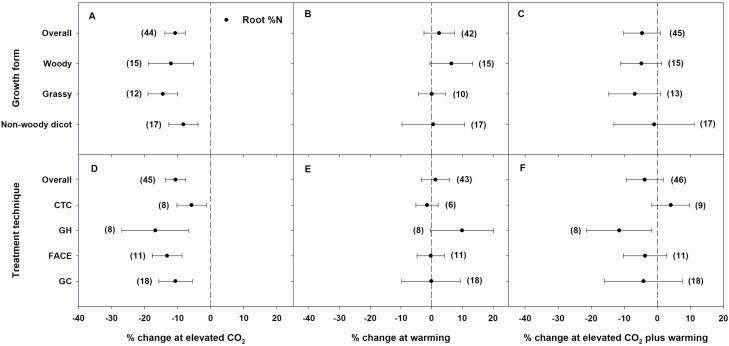
Percent change (compared to ambient or controls) in root %N in different growth forms (A–C) and elevated CO_2_ (eCO_2_) treatment techniques (D–F, CTC = closed-top chambers; GH = greenhouses; FACE = free-air CO_2_ enrichment; GC = growth chambers) in response to eCO_2_ (A, D), warming (B, E) and eCO_2_ plus warming (C, F). Each data point represents the mean ± 95 % CI. Numbers within parentheses represent the number of experimental observations. The dashed vertical line is the reference line at 0 % change. Treatment effects are non-significant at *P* < 0.05 if CIs overlap the zero line, and differences among treatments are non-significant if CIs overlap.

Elevated CO_2_ alone or in combination with warming significantly reduced shoot %N in all woody, grassy and non-woody dicot growth forms (Fig. 3A and C). Grasses had the smallest decrease in shoot %N in response to eCO_2_ (11 %) and to eCO_2_ plus warming (3 %), while non-woody dicots had the largest decrease in shoot %N in response to eCO_2_ (21 %) and eCO_2_ plus warming (19 %). Woody species had an intermediate decrease in shoot %N in response to eCO_2_ (19 %) and eCO_2_ plus warming (13 %) (Fig. 3A and C). Warming significantly increased shoot %N in both grasses (3 %) and non-woody dicots (5 %), while it non-significantly increased shoot %N in woody species (3 %) (Fig. 3B). Elevated CO_2_ (significantly, 12 % in woody; 15 % in grassy; and 8 % in non-woody dicot) and eCO_2_ plus warming (non-significantly, 5 % in woody; 7 % in grassy; and 1 % in non-woody dicot) reduced root %N in all three growth forms (Fig. 4A and C). Warming non-significantly increased root %N in woody species by 6 %, but it did not influence root %N in grasses or non-woody dicots (Fig. 4B).

Elevated CO_2_ alone or in combination with warming significantly decreased shoot %N in legumes (by 27 and 22 %, respectively) and non-legumes (16 and 12 %, respectively). However, warming by itself significantly increased shoot %N in non-legumes by 5 % but did not influence shoot %N of legumes (Fig. 3D–F).

In each eCO_2_ treatment technique, eCO_2_ significantly reduced both shoot and root %N, and this decrease was greatest for plants grown in GH (Figs 3G and 4D). Excluding GH, the magnitude of eCO_2_-driven decreases in shoot %N was similar across the different eCO_2_ techniques. Meanwhile, eCO_2_-driven decreases in root %N were similar for FACE (13 %) and GC (11%), both of which were similar to the overall decrease in root %N (11%). Except for plants grown in OTC, eCO_2_ plus warming significantly reduced shoot %N in plants grown using all eCO_2_ treatment techniques (Fig. 3I). Similar to eCO_2_ alone, this decrease in shoot %N was also greatest when plants were grown in GH. Likewise, similar decreases in shoot %N in response eCO_2_ plus warming were observed for plants grown using FACE (14 %), TGT (14 %) and GC (16 %), which were also similar to the overall decrease in shoot %N (14 %). A significant decrease in root %N in response to eCO_2_ plus warming was found only in plants grown in GH; all other treatment techniques were not significantly affected by eCO_2_ plus warming (Fig. 4F). Plants grown using FACE and growth-chamber techniques had similar decreases in root %N (4 %) in response to eCO_2_ plus warming which were similar to the overall decrease in root %N (3 %). Warming alone significantly (FACE and GC) or non-significantly (all other eCO_2_ treatment techniques) increased shoot %N, while, except for GH, warming did not influence root %N in plants grown using any other technique (Fig. 4E).

**Figure 5.**
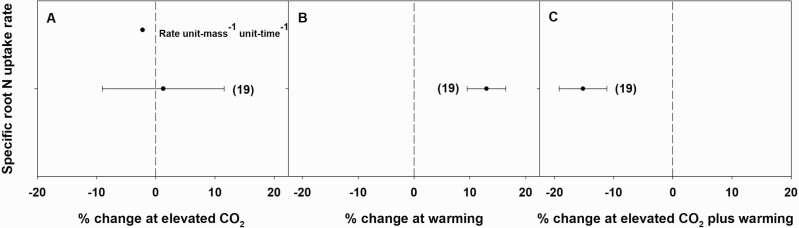
Percent change (compared to ambient or controls) in root N-uptake rate in response to elevated CO_2_ (A), warming (B) and elevated CO_2_ plus warming (C). Each data point represents the mean ± 95 % CI. Numbers within parentheses represent the number of experimental observations. The dashed vertical line is the reference line at 0 % change. Treatment effects are non-significant at *P* < 0.05 if CIs overlap the zero line.

Specific root N-uptake rate was not influenced by eCO_2,_ but it did significantly increase by 13 % in response to warming and significantly decrease by 15 % in response to eCO_2_ plus warming (Fig. 5). The publication bias could not be ignored for the individual effect of eCO_2_ on root N-uptake rate.

**Figure 6.**
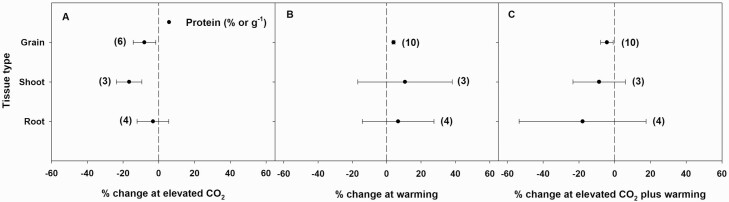
Percent change (compared to ambient or controls) in the concentration of total protein in different tissue types in response to elevated CO_2_ (A), warming (B) and elevated CO_2_ plus warming (C). Each data point represents the mean ± 95 % CI. Numbers within parentheses represent the number of experimental observations. The dashed vertical line is the reference line at 0 % change. Treatment effects are non-significant at *P* < 0.05 if CIs overlap the zero line, and differences among treatments are non-significant if CIs overlap.

Elevated CO_2_ alone or in combination with warming significantly or non-significantly decreased protein concentration in shoots, roots and grains, while warming alone significantly or non-significantly increased protein concentrations in all three tissue types (Fig. 6).

## Discussion

The effects of eCO_2_ on N concentration of plant tissues have been extensively studied and the findings often show a negative effect (Cotrufo *et al.* 1998; Curtis and Wang 1998; Ainsworth and Long 2005; Taub and Wang 2008). In agreement with previous reports, in this meta-analysis, eCO_2_ significantly reduced both shoot and root %N and the decrease was greater for shoots (17 %) than roots (10 %). These results are consistent with Cotrufo *et al.* (1998), who also found a 14 and 9 % decrease in above- and below-ground tissue N concentrations, respectively, in response to eCO_2_. As with the effects of eCO_2_ on %N, eCO_2_ plus warming also decreased %N in shoots (significantly) and roots (non-significantly). However, the magnitude of the negative effect of eCO_2_ plus warming on tissue %N was smaller than that of eCO_2_ alone. Previously, with smaller sample sizes, Zvereva and Kozlov (2006) and Wang *et al.* (2012) also reported negative effects of eCO_2_ plus warming on above-ground %N. The current meta-analysis further revealed that tissue quality (i.e. %N) can be persistently decreased with continuous exposure to eCO_2_, regardless of the temperature. Therefore, in the future, eCO_2_ is likely to reduce shoot %N regardless of the temperature, and, as a result, herbivores are likely to be N-limited and so would be required to consume more leaf tissues in order to meet their N requirement. This would eventually reduce photosynthetic rate followed by plant growth. One of the widely accepted hypotheses for low tissue %N at eCO_2_ is the dilution of N by increased photosynthetic assimilation of carbon (Ainsworth and Long 2005; Taub and Wang 2008). In addition, decreased root N-uptake rate has also been hypothesized as a potential cause of low tissue %N in plants grown at eCO_2_ (Taub and Wang 2008; Feng *et al.* 2015). Though the results of this meta-analysis do not support this hypothesis (Fig. 5A), it cannot be completely ruled out due to the large variation observed for N-uptake rate in response to eCO_2_ alone. However, in this meta-analysis, root N-uptake rates showed a positive relationship with shoot or root %N with warming or eCO_2_ plus warming, suggesting a greater dependence of N-uptake rate on temperature than CO_2_. Previously, Zvereva and Kozlov (2006), using 42 experimental observations, showed a negative but non-significant effect of warming on above-ground %N. In contrast, based on 119 experimental observations, the current meta-analysis reports a significant increase in above-ground %N in response to warming. Warming with <1.5 °C (*T*_L_) significantly increased shoot %N but this increase was neutralized as the magnitude of temperature elevation increased from *T*_L_ to *T*_M_ to *T*_H_ (<1.5, 1.5–5 or >5 °C above control), suggesting the inability of plants to maintain tissue quality (i.e. %N) at higher than optimal temperatures. Likewise, the magnitude of the negative effect of eCO_2_ plus warming on shoot %N further increased as temperature increased from *T*_L_ to *T*_M_, suggesting an inability of plants to maintain tissue quality at higher temperatures, even when combined with eCO_2_. *T*_M_ alone or in combination with eCO_2_ showed a tendency to increase root %N relative to the effect of *T*_L_ or its combination with eCO_2_ on root %N. However, *T*_H_ alone or in combination with eCO_2_ showed a tendency to decrease root %N relative to the effect of *T*_L_ or its combination with eCO_2_ on root %N. These results suggest that although shoot or root %N respond to different levels of CO_2_ similarly, irrespective of the temperature, they may not respond to different temperatures in a similar way, irrespective of the CO_2_ level.

Elevated CO_2_ is likely to reduce the protein concentration of many plant species, including those grown for human consumption. The C_3_ grasses such as wheat and rice are more likely to be negatively affected by eCO_2_ than legumes (Myers *et al.* 2014). Though the mechanism by which eCO_2_ decreases tissue protein concentration is not well understood, one possible explanation could be the increased concentration of non-structural carbohydrates relative to protein when plants are grown under eCO_2_ (Taub *et al.* 2008). In this meta-analysis, eCO_2_ alone or in combination with warming significantly or non-significantly reduced total protein concentrations in all shoots, roots and grains. These results are in conformity with the results observed for C_3_ grasses by Myers *et al.* (2014). Decreased protein concentration in edible portions of the crops can cause malnutrition among humans (Myers *et al.* 2014). In contrast, warming alone significantly or non-significantly increased the total protein concentration in all three tissue types. Interestingly, the variation in shoot or root protein concentration in response to eCO_2_ and/or warming scaled with the variation in shoot or root %N in response to these independent variables, suggesting a dependence of tissue protein concentration and, hence the nutritional quality, on tissue %N. At warming alone or eCO_2_ plus warming, tissue protein concentration also showed a positive relationship with root N-uptake rate, suggesting the dependence of nutritional quality on N-uptake rate when temperature is involved.

Grasses had the smallest decrease in shoot %N, but the largest decrease in root %N, in response to both eCO_2_ and eCO_2_ plus warming. In contrast, non-woody dicots had the largest decrease in shoot %N, but the smallest decrease in root %N, in response to both eCO_2_ and eCO_2_ plus warming. These results suggest a potential enhancement of net N translocation from roots-to-shoots in grasses, while a potential inhibition of net N translocation in non-woody dicots, in response to both eCO_2_ and eCO_2_ plus warming. Based on shoot and root %N data of woody species, both eCO_2_ and eCO_2_ plus warming are also likely to inhibit net N translocation in woody species. In addition, based on shoot and root %N data of grasses and non-woody dicots, warming is likely to enhance net N translocation in both growth forms. Collectively, these results suggest that the net translocation of N from roots-to-shoots will respond differently among plants of different growth forms to future climate conditions. Root-to-shoot translocation in woody species involves long-distance transportation compared to grasses or non-woody dicots. As eCO_2_ is likely to reduce xylem volume (Cohen *et al.* 2019), the observed decrease in net N translocation in woody species could be in part due to decreased xylem volume when plants grown at eCO_2_.

Legumes are known to have the ability to withstand the eCO_2_-driven leaf N dilution which is typically observed in C_3_ plants grown at eCO_2_ (Rogers *et al.* 2009). However, results of this meta-analysis oppose this view as eCO_2_ caused a greater decrease in legume shoot %N than non-legume shoot %N, regardless of the temperature. Notably, in this meta-analysis, a greater proportion of experimental observations of legume shoot %N were taken from studies which were conducted under natural soil nutrient conditions (FACE, OTC or TGT techniques with no additional nutrients supplied). As van Groenigen *et al.* (2006) explained, eCO_2_ will not have an effect on N_2_ fixation when legumes are grown under natural conditions with no fertilizer additions, and this could be one of the potential reasons for the observed result in this study. Meanwhile, though warming significantly increased non-legume shoot %N, it did not have an effect on legume shoot %N. As Hungria and Kaschuk (2014) explained, warming can limit N_2_ fixation by inhibiting NH_4_^+^ assimilation and nitrogenase activity, which could be one of the potential reasons for the observed neutral effect of warming on shoot %N.

In this meta-analysis, a subgroup analysis of different treatment techniques was conducted to find those more suitable for climate studies involving the interaction of CO_2_ and temperature. All of these techniques have their own advantages and disadvantages. Both shoot and root %N were investigated here because, apart from biomass measures, these have been widely measured in plants grown with these treatment techniques. Since this meta-analysis focused primarily on the effects of eCO_2_ plus warming on plant N relations, the suitability of these techniques is mainly discussed in response to eCO_2_ plus warming. The FACE technique is thought to provide the most realistic measure of the effects of eCO_2_ on crop yields because enclosure techniques can produce a ‘chamber effect’ that can exceed the effects of eCO_2_ (Ainsworth *et al.* 2005, 2008; Ainsworth and Long 2021). However, in this meta-analysis, some enclosure studies produced results similar to those of FACE studies (e.g. shoot %N in TGT and root %N in GC). Previously, a meta-analysis conducted by Taub *et al.* (2008) also reported similar protein concentrations in response to eCO_2_ in crops grown using either FACE or other techniques (OTC, CTC, GH, GC). In the current meta-analysis, the overall decreases in shoot and root %N in response eCO_2_ plus warming were 14 and 3 %, respectively. Interestingly, FACE and TGT studies also showed 14 % decreases in shoot %N, and GC and CTC studies showed 16 and 11 % decreases in shoot %N, respectively, in response to eCO_2_ plus warming. Meanwhile, the 4 % decrease in root %N observed for FACE and GC was similar to the overall decrease in root %N in response to eCO_2_ plus warming. These results suggest that in addition to FACE technique, other enclosed techniques such as GC, TGT and CTC can produce reliable results when studying the effects of eCO_2_ plus warming. Additionally, these three enclosed techniques produced similar results to those of the FACE technique in response to warming alone. This meta-analysis further suggests that the GH technique is likely unsuitable for studies involved with eCO_2_ plus warming, due to its overestimation of the negative impacts of eCO_2_ plus warming on plant %N.

In the future, global environmental changes such as CO_2_ enrichment, warming, drought, N deposition, etc. will occur concomitantly. Therefore, multi-factor manipulation approaches will be necessary to understand the combined effects of these various factors on plant growth, metabolism and production. This meta-analytic review was designed to improve understanding of the effects of eCO_2_ plus warming on plant N metabolism as this area of research has important knowledge gaps. However, one of the limitations of this study was the analysis of the effects of only two predictor variables on plant N metabolism-related response variables. Therefore, future research should focus on incorporating more predictor variables, such as drought, when investigating the impacts of environmental change on plant N metabolism. In addition, it will be interesting to see how experimental duration and the level and form of N affect plant N metabolism under these conditions, and how plant N metabolism-related variables respond to different levels of CO_2_ and temperature.

## Conclusions

In the future, concomitant increases in CO_2_ and temperature are likely to affect plant N metabolism by lowering plant %N, root N-uptake rate and tissue protein concentration. Therefore, when developing plants for future climates, plant-improvement efforts should focus on generating new genotypes with more-resilient N metabolism.

## Supporting Information

The following additional information is available in the online version of this article—

**Appendix S1.** Database.

**Appendix S2.** References included in the meta-analysis.

plab031_suppl_Supplementary_Materials

## Data Availability

The data are available as **Supporting Information**.

## Notes

**Plants, Ecosystems & Climate. Chief Editor:** Mary Heskel
