# Supplementary material for: A meta-analysis of the combined effects of elevated carbon dioxide and chronic warming on plant %N, protein content and N-uptake rate
Source: AoB Plants. 2021 May 25;13(4):plab031. doi: 10.1093/aobpla/plab031 (PMC8286714; doi:10.1093/aobpla/plab031)
Supplement: plab031_suppl_Supplementary_Materials [file plab031_suppl_supplementary_materials.zip › plab031_suppl_Supporting Information-Appendix_S2_References_included_in_the_meta_analysis.pdf]

**Appendix S2: References included in the meta-analysis**

Abebe A, Pathak H, Singh SD, Bhatia A, Harit RC, Kumar V. 2016. Growth, yield and quality of maize with elevated atmospheric carbon dioxide and temperature in north-west India. *Agriculture, Ecosystems & Environment* 218:66-72.

Anderson LJ, Cipollini D. 2013. Gas exchange, growth, and defense responses of invasive *Alliaria petiolata* (Brassicaceae) and native *Geum vernum* (Rosaceae) to elevated atmospheric $CO_2$ and warm spring temperatures. *American Journal of Botany* 100:1544-1554.

Andresen LC, Michelsen A, Ambus P, Beier C. 2010. Belowground heathland responses after 2 years of combined warming, elevated $CO_2$ and summer drought. *Biogeochemistry* 101:27-42.

Andresen LC, Michelsen A, Jonasson S, Beier C, Ambus P. 2009. Glycine uptake in heath plants and soil microbes responds to elevated temperature, $CO_2$ and drought. *Acta Oecologica* 35:786-796.

Aranjuelo I, Irigoyen JJ, Sánchez-Díaz M, Nogués S. 2008. Carbon partitioning in $N_2$ fixing *Medicago sativa* plants exposed to different $CO_2$ and temperature conditions. *Functional Plant Biology* 35:306-317.

Aranjuelo I, Pérez P, Hernández L, Irigoyen JJ, Zita G, Martínez-Carrasco R, Sánchez-Díaz M. 2005. The response of nodulated alfalfa to water supply, temperature and elevated $CO_2$: photosynthetic downregulation. *Physiologia Plantarum* 123:348-358.

Ariz I, Cruz C, Neves T, Irigoyen J, García C, Nogués S, Aparicio-Tejo PM, Aranjuelo I. 2015. Leaf $\delta^{15}N$ as a physiological indicator of the responsiveness of $N_2$-fixing alfalfa plants to elevated [$CO_2$], temperature and low water availability. *Frontiers in Plant Science* 6:574.

Arndal MF, Schmidt IK, Kongstad J, Beier C, Michelsen A. 2014. Root growth and N dynamics in response to multi-year experimental warming, summer drought and elevated $CO_2$ in a mixed heathland-grass ecosystem. *Functional Plant Biology* 41:1-10.

Ayub G, Smith RA, Tissue DT, Atkin OK. 2011. Impacts of drought on leaf respiration in darkness and light in *Eucalyptus saligna* exposed to industrial-age atmospheric $CO_2$ and growth temperature. *New Phytologist* 190:1003-1018.

Chen H, Rygiewicz PT, Johnson MG, Harmon ME, Tian H, Tang JW. 2008. Chemistry and long-term decomposition of roots of Douglas-fir grown under elevated atmospheric carbon dioxide and warming conditions. *Journal of Environmental Quality* 37:1327-1336.

Coleman JS, Bazzaz FA. 1992. Effects of $CO_2$ and temperature on growth and resource use of co-occurring $C_3$ and $C_4$ annuals. *Ecology* 73:1244-1259.

Crous KY, Quentin AG, Lin YS, Medlyn BE, Williams DG, Barton CVM, Ellsworth DS. 2013. Photosynthesis of temperate Eucalyptus globulus trees outside their native range has limited adjustment to elevated $CO_2$ and climate warming. *Global Change Biology* 19:3790-3807.

de Assis Prado CHB, de Camargo-Bortolin LHG, Castro É, Martinez CA. 2016. Leaf dynamics of Panicum maximum under future climatic changes. *PLoS ONE* 11:e0149620.

Dijkstra FA, Blumenthal D, Morgan JA, Pendall E, Carrillo Y, Follett RF. 2010. Contrasting effects of elevated $CO_2$ and warming on nitrogen cycling in a semiarid grassland. *New Phytologist* 187:426-437.

Dury SJ, Good JEG, Perrins CM, Buse A, Kaye T. 1998. The effects of increasing $CO_2$ and temperature on oak leaf palatability and the implications for herbivorous insects. *Global Change Biology*, 4:55-61.

Ghannoum O, Phillips NG, Conroy JP, Smith RA, Attard RD, Woodfield R, Logan BA, Lewis JD, Tissue DT. 2010a. Exposure to preindustrial, current and future atmospheric $CO_2$ and temperature differentially affects growth and photosynthesis in *Eucalyptus*. *Global Change Biology* 16:303-319.

Ghannoum O, Phillips NG, Sears MA, Logan BA, Lewis JD, Conroy JP, Tissue DT. 2010b. Photosynthetic responses of two eucalypts to industrial-age changes in atmospheric [$CO_2$] and temperature. *Plant, Cell & Environment* 33:1671-1681.

Gherlenda AN, Haigh AM, Moore BD, Johnson SN, Riegler M. 2015. Responses of leaf beetle larvae to elevated [$CO_2$] and temperature depend on *Eucalyptus* species. *Oecologia* 177:607-617.

Gherlenda AN, Haigh AM, Moore BD, Johnson SN, Riegler M. 2016. Climate change, nutrition and immunity: effects of elevated $CO_2$ and temperature on the immune function of an insect herbivore. *Journal of insect physiology* 85:57-64.

Hobbie EA, Olszyk DM, Rygiewicz PT, Tingey DT, Johnson MG. 2001. Foliar nitrogen concentrations and natural abundance of $^{15}N$ suggest nitrogen allocation patterns of Douglas-fir and mycorrhizal fungi during development in elevated carbon dioxide concentration and temperature. *Tree Physiology* 21:1113-1122.

Jauregui I, Aroca R, Garnica M, Zamarreño ÁM, García-Mina JM, Serret MD, Parry M, Irigoyen JJ, Aranjuelo I. 2015. Nitrogen assimilation and transpiration: key processes conditioning responsiveness of wheat to elevated [$CO_2$] and temperature. *Physiologia Plantarum* 155: 338-354.

Jayawardena DM, Heckathorn SA, Bista DR, Mishra S, Boldt JK, Krause CR. 2017. Elevated $CO_2$ plus chronic warming reduce nitrogen uptake and levels or activities of nitrogen-uptake and -assimilatory proteins in tomato roots. *Physiologia Plantarum* 159:354-365.

Jayawardena DM, Heckathorn SA, Boldt JK. 2020. Effects of elevated carbon dioxide and chronic warming on nitrogen (N)-uptake rate, -assimilation, and -concentration of wheat. *Plants* 9:1689.

Jayawardena DM, Heckathorn SA, Rajanayake KK, Boldt JK, Isailovic D. 2021. Elevated carbon dioxide and chronic warming together decrease nitrogen uptake rate, net translocation, and assimilation in tomato. *Plants* 10:722.

Jing L, Wang J, Shen S, Wang Y, Zhu J, Wang Y, Yang L. 2016. The impact of elevated $CO_2$ and temperature on grain quality of rice grown under open-air field conditions. *Journal of the Science of Food and Agriculture* 96:3658-3667.

Johns CV, Beaumont LJ, Hughes L. 2003. Effects of elevated $CO_2$ and temperature on development and consumption rates of *Octotoma championi* and *O. scabripennis* feeding on *Lantana camara*. *Entomologia Experimentalis et Applicata* 108:169-178.

Johns CV, Hughes L. 2002. Interactive effects of elevated $CO_2$ and temperature on the leaf-miner *Dialectica scalariella* Zeller (Lepidoptera: Gracillariidae) in Paterson's Curse, *Echium plantagineum* (Boraginaceae). *Global Change Biology* 8:142-152.

Johnson SN, Hartley SE. 2018. Elevated carbon dioxide and warming impact silicon and phenolic-based defences differently in native and exotic grasses. *Global Change Biology* 24:3886-3896.

Kandeler E, Tscherko D, Bardgett RD, Hobbs PJ, Kampichler C, Jones TH. 1998. The response of soil microorganisms and roots to elevated $CO_2$ and temperature in a terrestrial model ecosystem. *Plant and Soil* 202:251-262.

Kellomäki S, Wang KY. 2001. Growth and resource use of Birch seedlings under elevated carbon dioxide and temperature. *Annals of Botany* 87:669-682.

Kim HY, Lim SS, Kwak JH, Lee DS, Lee SM, Ro HM, Choi WJ. 2011. Dry matter and nitrogen accumulation and partitioning in rice (*Oryza sativa* L.) exposed to experimental warming with elevated $CO_2$. *Plant and Soil* 342:59-71.

Kim SH, Gitz DC, Sicher RC, Baker JT, Timlin DJ, Reddy VR. 2007. Temperature dependence of growth, development, and photosynthesis in maize under elevated $CO_2$. *Environmental and Experimental Botany* 61:224-236.

King JS, Thomas RB, Strain BR. 1997. Morphology and tissue quality of seedling root systems of *Pinus taeda* and *Pinus ponderosa* as affected by varying $CO_2$, temperature, and nitrogen. *Plant and Soil* 195:107-119.

Kuokkanen K, Julkunen-Tiitto R, Keinänen M, Niemelä P, Tahvanainen J. 2001. The effect of elevated $CO_2$ and temperature on the secondary chemistry of *Betula pendula* seedlings. *Trees* 15:378-384.

Kuokkanen K, Yan S, Niemelä P. 2003. Effects of elevated $CO_2$ and temperature on the leaf chemistry of birch *Betula pendula* (Roth) and the feeding behaviour of the weevil *Phyllobius maculicornis*. *Agricultural and Forest Entomology* 5:209-217.

Lavola A, Nybakken L, Rousi M, Pusenius J, Petrelius M, Kellomäki S, Julkunen-Tiitto R. 2013. Combination treatment of elevated UVB radiation, $CO_2$ and temperature has little effect

on silver birch (*Betula pendula*) growth and phytochemistry. *Physiologia Plantarum* 149:499-514.

Li C, Zhu JG, Sha LN, Zhang JS, Zeng Q, Liu G. 2017. Rice (*Oryza sativa* L.) growth and nitrogen distribution under elevated $CO_2$ concentration and air temperature. *Ecological research* 32:405-411.

Lilley JM, Bolger TP, Peoples MB, Gifford RM. 2001. Nutritive value and the nitrogen dynamics of *Trifolium subterraneum* and *Phalaris aquatica* under warmer, high $CO_2$ conditions. *New Phytologist* 150:385-395.

Liu Y, Dang Z, Wang Y, Parajulee MN, Chen F. 2019. Interactive Effects of [$CO_2$] and temperature on plant chemistry of transgenic Bt Rice and population dynamics of a non-target planthopper, *Nilaparvata lugens* (Stål) under different levels of soil nitrogen. *Toxins* 11:261.

Luomala EM, Laitinen K, KellomÄKi S, Vapaavuori E. 2003. Variable photosynthetic acclimation in consecutive cohorts of Scots pine needles during 3 years of growth at elevated $CO_2$ and elevated temperature. *Plant, Cell & Environment* 26:645-660.

Mueller KE, Blumenthal DM, Pendall E, Carrillo Y, Dijkstra FA, Williams DG, Follett RF, Morgan JA. 2016. Impacts of warming and elevated $CO_2$ on a semi-arid grassland are non-additive, shift with precipitation, and reverse over time. *Ecology Letters* 19:956-966.

Murray TJ, Tissue DT, Ellsworth DS, Riegler M. 2013. Interactive effects of pre-industrial, current and future [$CO_2$] and temperature on an insect herbivore of *Eucalyptus*. *Oecologia* 171:1025-1035.

Norby RJ, Long TM, Hartz-Rubin JS, O'Neill EG. 2000. Nitrogen resorption in senescing tree leaves in a warmer, $CO_2$-enriched atmosphere. *Plant and Soil* 224:15-29.

Palacios CJ, Grandis A, Carvalho VJ, Salatino A, Buckeridge MS. 2019. Isolated and combined effects of elevated $CO_2$ and high temperature on the whole-plant biomass and the chemical composition of soybean seeds. *Food chemistry* 275:610-617.

Prasad PVV, Boote KJ, Vu JCV, Allen Jr LH. 2004. The carbohydrate metabolism enzymes sucrose-P synthase and ADG-pyrophosphorylase in phaseolus bean leaves are up-regulated at elevated growth carbon dioxide and temperature. *Plant Science* 166:1565-1573.

Qiao Y, Miao S, Li Q, Jin J, Luo X, Tang C. 2019. Elevated $CO_2$ and temperature increase grain oil concentration but their impacts on grain yield differ between soybean and maize grown in a temperate region. *Science of The Total Environment* 666:405-413.

Ramalho JC, Pais IP, Leitão AE, Guerra M, Reboredo FH, Máguas CM, Carvalho ML, Scotto-Campos P, Ribeiro-Barros AI, Lidon FJC, DaMatta FB. 2018. Can elevated air [$CO_2$] conditions mitigate the predicted warming impact on the quality of coffee bean? *Frontiers in Plant Science* 9:287.

Rosenthal DM, Ruiz-Vera UM, Siebers MH, Gray SB, Bernacchi CJ, Ort DR. 2014. Biochemical acclimation, stomatal limitation and precipitation patterns underlie decreases in photosynthetic stimulation of soybean (*Glycine max*) at elevated [$CO_2$] and temperatures under fully open air field conditions. *Plant Science* 226:136-146.

Salazar-Parra C, Aranjuelo I, Pascual I, Erice G, Sanz-Sáez Á, Aguirreolea J, Sánchez-Díaz M, Irigoyen JJ, Araus JL, Morales F. 2015. Carbon balance, partitioning and photosynthetic acclimation in fruit-bearing grapevine (*Vitis vinifera* L. cv. Tempranillo) grown under simulated climate change (elevated $CO_2$, elevated temperature and moderate drought) scenarios in temperature gradient greenhouses. *Journal of Plant Physiology* 174:97-109.

Seth CS, Misra V. 2014. Changes in C-N metabolism under elevated $CO_2$ and temperature in Indian mustard (*Brassica juncea* L.): an adaptation strategy under climate change scenario. *Journal of Plant Research* 127:793-802.

Sharwood RE, Crous KY, Whitney SM, Ellsworth DS, Ghannoum O. 2017. Linking photosynthesis and leaf N allocation under future elevated $CO_2$ and climate warming in *Eucalyptus globulus*. *Journal of Experimental Botany* 68:1157-1167.

Soussana JF, Casella E, Loiseau P. 1996. Long-term effects of $CO_2$ enrichment and temperature increase on a temperate grass sward. *Plant and Soil* 182:101-114.

Veteli TO, Kuokkanen K, Julkunen-Tiitto R, Roininen H, Tahvanainen J. 2002. Effects of elevated $CO_2$ and temperature on plant growth and herbivore defensive chemistry. *Global Change Biology* 8:1240-1252.

Volder A, Gifford RM, Evans JR. 2015. Effects of elevated atmospheric $CO_2$ concentrations, clipping regimen and differential day/night atmospheric warming on tissue nitrogen concentrations of a perennial pasture grass. *AoB Plants* 7.

Wan S, Norby RJ, Pregitzer KS, Ledford J, O'Neill EG. 2004. $CO_2$ enrichment and warming of the atmosphere enhance both productivity and mortality of maple tree fine roots. *New Phytologist* 162:437-446.

Williams RS, Lincoln DE, Norby RJ. 2003. Development of gypsy moth larvae feeding on red maple saplings at elevated $CO_2$ and temperature. *Oecologia* 137:114-122.

Williams RS, Norby RJ, Lincoln DE. 2000. Effects of elevated $CO_2$ and temperature-grown red and sugar maple on gypsy moth performance. *Global Change Biology* 6:685-695.

Zavalloni C, Vicca S, Büscher M, de la Providencia IE, Dupré de Boulois H, Declerck S, Nijs I, Ceulemans R. 2012. Exposure to warming and $CO_2$ enrichment promotes greater above-

ground biomass, nitrogen, phosphorus and arbuscular mycorrhizal colonization in newly established grasslands. *Plant and Soil* 359:121-136.

Zhang S, Fu W, Zhang Z, Fan Y, Liu T. 2017. Effects of elevated $CO_2$ concentration and temperature on some physiological characteristics of cotton (*Gossypium hirsutum* L.) leaves. *Environmental and Experimental Botany* 133:108-117.
